# Monitoring Water Stress in Grapevine (*Vitis vinifera* L.) Using Proximal Hyperspectral Imaging

**DOI:** 10.3390/plants15091372

**Published:** 2026-04-30

**Authors:** Jon Ruiz-de-Gauna, Silvia Arazuri, Patricia Viela, Maider Velaz, Sara León-Ecay, Carmen Jarén, Ainara López-Maestresalas

**Affiliations:** 1 Institute on Innovation and Sustainable Development in Food Chain (IS-FOOD), Department of Engineering, Universidad Pública de Navarra (UPNA), Campus de Arrosadia, 31006 Pamplona, Spain; jon.ruizdegauna@unavarra.es (J.R.-d.-G.); silvia.arazuri@unavarra.es (S.A.); patricia.viela@unavarra.es (P.V.); sara.leon@unavarra.es (S.L.-E.); cjaren@unavarra.es (C.J.); 2 Institute for Multidisciplinary Research in Applied Biology (IMAB), Department of Agronomy, Biotechnology and Food, Universidad Pública de Navarra (UPNA), Campus de Arrosadia, 31006 Pamplona, Spain; maider.velaz@unavarra.es

**Keywords:** *Vitis vinifera* L., stem water potential, early detection, chemometrics, VIS-NIR, red-edge, PCA, Mahalanobis distance, time series

## Abstract

This study addresses the early detection of water stress in grapevines (*Vitis vinifera* L. cv. Monastrell), a key challenge for precision irrigation. The main objective is to assess the feasibility of VIS–NIR hyperspectral imaging (400–1000 nm) to anticipate water stress, relating the spectral signal to stem water potential. This study was developed over two campaigns, in 2024 and 2025, using 18 potted plants. In 2024, eight vines were irrigated, and the remaining 10 were subjected to water-deprivation treatments, whilst in 2025, all plants were irrigated, but half at a control dose and the rest at a reduced dose equivalent to 33% of the control. Images were acquired over five dates in June 2024 and over seven in June 2025 using a Specim IQ camera; stem potential was also measured to provide a physiological reference. Individual time series were developed, calculating the Mahalanoubis distance in a PCA space. Results revealed a change window between 10 and 13 June, consistent with the divergence in water potential from 17 to 24 June. PCA highlighted spectral regions related to changes in pigments, nitrogen and water content as main indicators of water stress. We conclude that HSI is a promising tool for early water stress detection.

## 1. Introduction

The vine is a climbing plant belonging to the *Vitis vinifera* L. species, which is used, almost exclusively, as a crop for grape and wine production [1]. Like any other crop, it can be affected by various biotic and abiotic factors that can harm crop yield and the quality of the final product. Biotic factors include organisms that cause diseases and pests in grapevines (e.g., fungi, bacteria, viruses, and insects), while abiotic factors consist of environmental factors that can influence crop development, such as temperature, wind and water and nutrient availability [2,3,4,5,6,7].

Water stress is an abiotic factor defined as a physiological condition in which the plant is subjected to an excess or insufficiency of water in its environment. In the case of water deficit, it can be described as a situation in which a lack of moisture exists, making it impossible for the vine to satisfy its physiological needs. This condition is influenced by external factors such as climate, soil type (water retention capacity) and irrigation (amount and frequency) [2,4,8,9].

Water stress has been demonstrated to elicit a series of responses in plants. Furthermore, in regions characterized by recurrent water shortages, plants have evolved specific physiological and biochemical mechanisms to enable survival and adaptation. In the case of grapevines, the primary mechanisms comprise reduced vegetative growth due to decreased turgor pressure in the cells, deeper root growth (although there is a decrease in total root mass), and reduced photosynthetic activity, which affects biomass production [2,10,11]. In relation to the fruit, severe stress has been shown to have a substantial impact on the quantity and size of the grapes, as well as causing undesirable changes in their composition. Nevertheless, moderate stress during stages, such as ripening, helps to increase the concentration of compounds of interest (sugars, acids, phenolic compounds, etc.), which can improve the quality of the final product [11].

Therefore, it is necessary to devise methods that enable effective monitoring of the crop water status so that stress situations can be detected and corrected as soon as possible, minimizing the damage they cause. In this context, the use of optical sensors, such as hyperspectral imaging technology, emerges as a noteworthy instrument. The rationale behind this is that it facilitates the acquisition of information from the sample in situ, with no or minimal preparation, in a rapid, non-invasive and non-destructive manner [12]. Hyperspectral imaging (HSI) is a technique that combines the advantages of conventional near-infrared spectroscopy (NIRS) and imaging. It generates a three-dimensional hypercube image, with the first two dimensions providing spatial information and the third dimension providing spectral information related to the composition of the sample [12,13].

HSI technology has already been tested for plant diseases and pests monitoring as well as for water stress modeling. Studies such as those conducted by Loggenberg et al. and Zodvko et al. [13,14], which attempt to identify vines under water stress using classification methods like Random Forest (RF) and Partial Least Squares Discriminant Analysis (PLS-DA) combined with Support Vector Machines (SVMs), show a correct performance with accuracies exceeding 80% and 90%, respectively. With regard to disease and pest monitoring, it is quite common for experiments to be carried out at leaf or canopy level, with the most common sensors being those operating in the visible and near-infrared ranges [15]. Examples of studies related to pests and diseases include those by Swaraj & Aparma, Nguyen et al. and Pérez-Roncal et al. [7,16,17], which obtained accuracies ranging from 60% to 90%.

The lack of established literature on in situ monitoring and early water stress detection in vineyards highlights the need to explore and validate the potential of hyperspectral imaging technology for this purpose.

Therefore, the objective of this study was to evaluate the feasibility of using a proximal hyperspectral imaging system for the in-field detection of water stress in grapevine leaves of the cv. Monastrell. To this end, the selected vines were monitored during the development of water stress. Then, using the PCA method together with the Mahalanobis distance calculation, a comparison was made between the data corresponding to the first day of the experiment and those of the following days. In this way, it was observed that the method applied is quite useful for detecting early symptoms corresponding to water stress.

## 2. Results

### 2.1. Stem Water Potential Reference Values 

Table 1 shows the mean value and standard deviation of the stem potential obtained on each of the measurement days, both for vines subjected to water stress and for the control (irrigated) ones. For the 2024 campaign, it can be observed how, during the first two days of measurement, the potential values remained fairly similar between both groups (−0.62 MPa versus −0.64 MPa for the 3rd of June and −0.39 MPa versus −0.42 MPa for the 10th of June). However, from the third day of measurement onwards, noticeable differences began to appear between the stressed group and the control group, which remained until the end of the test (−0.67 MPa versus −0.42 MPa for the 17th of June, −0.71 MPa versus −0.41 MPa for the 21st of June and −0.90 MPa versus −0.61 MPa for the 24th of June). In the case of the 2025 campaign, values remained similar during the first two days (−0.56 MPa versus −0.54 MPa for the 9th of June and −0.70 MPa versus −0.64 MPa for the 16th of June), and differences appeared on the third day (−0.90 MPa versus −0.58 MPa for the 24th of June). It can be stated that, regarding the observed values, the water stress developed by the plants can be considered moderate stress. In order to consider a high degree of water stress, the observed values should be below −0.90 MPa or even −1.00 MPa.

Table 1 also reflects the results of the ANOVA analyses for each day, comparing the stem water potential values between the stress and control groups to determine whether or not there were significant differences. It can be seen that, for both campaigns, during the first two days of measurement (3rd and 10th of June for 2024 and 9th and 16th of June for 2025) the differences between groups were not significant, as the *p*-values were equal to 0.275 and 0.538 for the year 2024 and to 0.603 and 0.136 for 2025. Nevertheless, from the third day of measurement onwards (17th of June for 2024 and 24th of June for 2025), these differences became significant as the analysis presented *p*-values below 0.01.

### 2.2. Hyperspectral Imaging

#### 2.2.1. Spectral Profile

**Figure 1:**
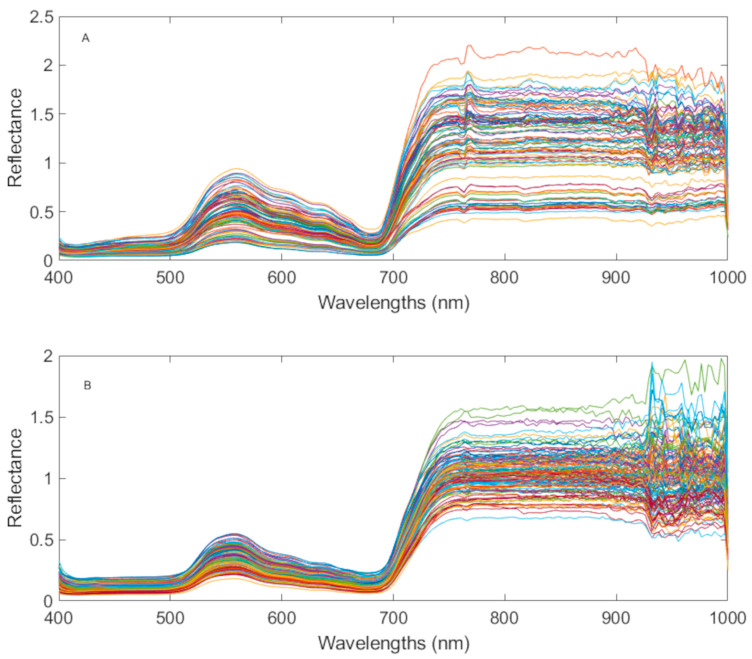
Average raw reflectance spectra for the years 2024 (**A**) and 2025 (**B**).

Figure 1A,B presents the average raw reflectance spectra per vine and day for the two years. The spectrum showed a clear pattern in both cases, with two valley areas that are well differentiated. The first valley is located between 400 and 500 nm, while the second is situated approximately at 700 nm. According to Workman & Weyer [18], spectra at 500 nm are associated with the vibration of O-H groups with no hydrogen bonding (R-C-OH), whilst the absorbance at 700 nm is associated with C-H aromatic groups (ArCH). This statement is consistent with the effects generated as a result of the presence of photosynthetic pigments, such as chlorophyll, anthocyanins and carotenoids. Those pigments contain the aforementioned groups in their composition [19,20]; it is therefore logical that they absorb a significant amount of radiation at 500 nm and 700 nm, giving rise to the observed reflectance valleys. The final range, from 925 nm to 1000 nm, presented very pronounced oscillations (spectral noise). Therefore, generating difficulty for interpreting the spectrum. In the case of the year 2024 (Figure 1A), this noise was not excessively high, so a proper correction was expected by means of pre-treatment. Only the range from 980 to 990 nm to 1000 nm reflected excessive noise, so it was removed before data analysis. As for the 2025 spectra (Figure 1B), it was observed that the noise was very high, with a clear difference when compared to 2024. Consequently, for the 2025 data, the whole final range was removed (from 926 nm to 1000 nm).

Differences in reflectance levels were observed between the two years. This may be due to variations in the lighting conditions of the environment, something that can occur from one year to the next, as the images were taken in natural light conditions.

#### 2.2.2. Change Day Obtained by PCA and Mahalanobis Distance

As described in Section 4.4.2, a principal component analysis (PCA) was carried out individually on the data for each vine. For the year 2024, throughout the 18 PCA analyses, the variance explained by the first principal component (PC1) ranged between 63% and 93%. For the year 2025, explained variance by PC1 varied from 54% to 91%. In most cases, PC1 explained more than 70% of the variance. Likewise, in 2024, the explained variance by PC2 and PC3 ranged between 3–30% and 1–7%, respectively. Similarly, in 2025, the explained variance ranges were 3–30% and 0.8–8% for PC2 and PC3, respectively.

**Figure 2:**
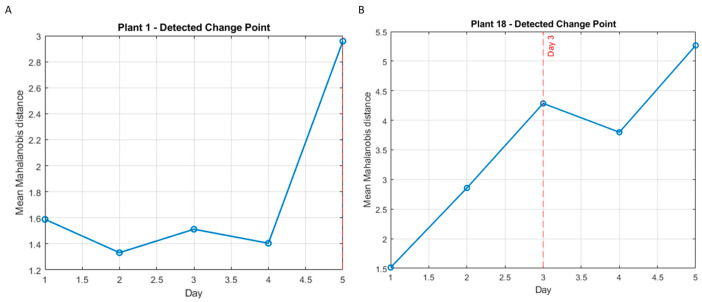
Evolution of mean Mahalanobis distance and detected change point for the vine (*Vitis vinifera* L. cv. Monastrell) A06 110R (control group) (**A**) and for the vine B15 140R (stress group) (**B**).

Figure 2A,B shows two examples of the evolution of the Mahalanobis distance throughout the trial of the 2024 campaign, the first corresponding to a vine from the control group (Figure 2A) and the second to a plant from the stress group (Figure 2B). As can be observed, for the control plant, there was no significant change until day 5, (red dashed line) while for the stressed vine, this change was clearly noticed on day 3 (red dashed line). This can be considered as a consistent result, since the group in which the greatest change was expected at an earlier stage was the stress group.

**Figure 3:**
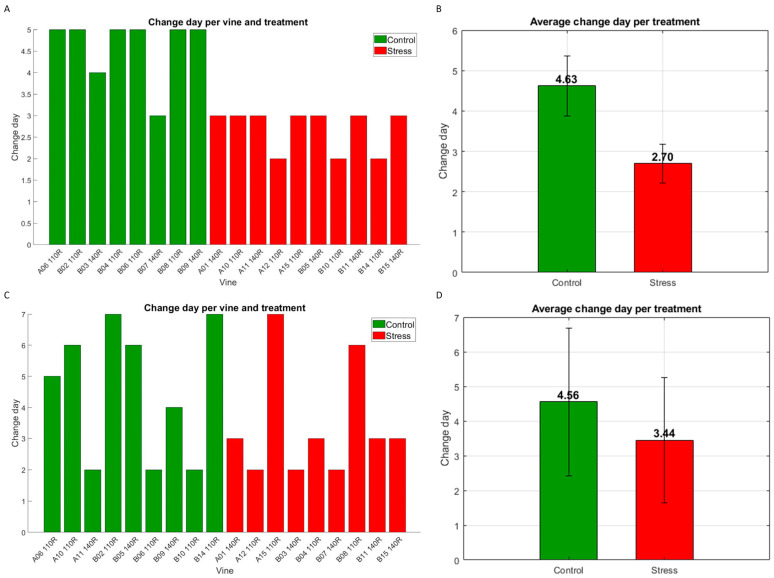
Detected change day per vine (*Vitis vinifera* L. cv. Monastrell) (**A**) and average change day per group/treatment (**B**) in 2024; detected change day per vine (**C**) and average change day per group/treatment (**D**) in 2025.

Figure 3 reflects the assigned change day for each vine (A,C), as well as the group average and standard deviation (B,D) for each year. Focusing on the behavioral patterns observed for each group, in 2024 the earlier change for the stressed vines is clearly noticeable. In the control group, all plants were assigned day 5, except for the vines B03 140R and B07 140R that changed on days 4 and 3, respectively, whereas the change day for the stress group was assigned to either day 2 or 3 in all cases (Figure 3A). The average between groups also revealed that control vines did not follow the same behavioral pattern as the stressed vines. The average day of change in the control group was 4.63 compared to 2.7 in the stress group (Figure 3B). On the other hand, in 2025 the behavioral pattern remains consistent, as an earlier change is perceived in the stress group, but in a less clear way. In this case, the control group contained four plants with early change days (A11 140R, B06 110R and B10 110R on day 2 and B09 140R on day 4) and only two vines (B02 110R and B14 110R) reached day 7. In the stress group, there were two plants with no or late changes (A15 110R on day 7 and B08 110R on day 6), while the rest of the vines showed variations on day 2 or 3 (Figure 3C). The average day of change was 4.56 for the control group and 3.44 for the stress group (Figure 3D). As for the rootstocks, a clear behavioral pattern has not been perceived for vines corresponding to the ‘140 Ruggeri’ rootstock nor for the ‘110 Richter’ ones.

The difference observed between the results of 2024 and 2025 is consistent when considering, first of all, the change in procedure between the trials of both campaigns. It should be noted that in 2024, irrigation was completely interrupted for the plants in the stress group, whereas in 2025, the irrigation dose was reduced, but without completely eliminating the water supply. Thus, it is logical to assume that the water stress developed in 2025 was less severe and, therefore, the differences between control and stress groups were smaller compared to 2024. Another factor to consider is the intrinsic variability of the plant. In other words, each vine, being a living organism, undergoes changes over time (growth, development of vegetative parts and reproductive organs, etc.), and, although trials have been designed to reduce this effect as much as possible, spectral data can still be affected. In this case, it is clear that intrinsic variation had a higher effect on the results of 2025. Nevertheless, the general trend observed in both years was that vines subjected to water stress showed a greater and earlier tendency to change, something consistent with what was expected.

Furthermore, when comparing the results with the measurement of stem water potential (Table 1), it is clear that in most cases, the changes in the stress group were detected earlier by spectral analysis. This is because, in the spectral results, most stressed plants showed the point of change on day 2 or 3 (7th and 10th of June in 2024 and 11th and 13th of June in 2025), while, when using stem potential data, no significant differences were observed until the 17th of June in 2024 and the 24th of June in 2025.

#### 2.2.3. Spectral Ranges of Importance

**Figure 4:**
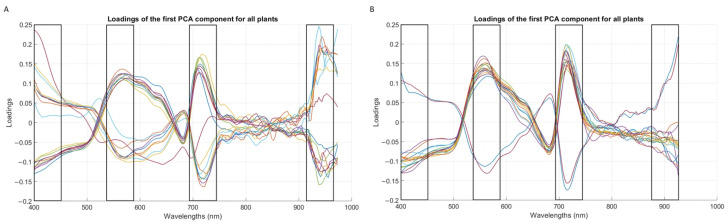
Loadings plot for 2024 (**A**) and 2025 (**B**).

Figure 4 presents the loadings plots, which represent the relative importance of each wavelength for the first principal component. Each line represents the loadings corresponding to one of the 18 PCAs (one for each plant and year). In both 2024 (Figure 4A) and 2025 (Figure 4B), the most important spectral ranges have been highlighted. It can be seen that the two plots followed a very similar pattern, both presenting the highest values and, therefore, the most significant spectral information in the ranges of 400–450 nm, 540–590 nm and 690–740 nm. The effect in these ranges is attributed to the action of photosynthetic pigments, as already explained in the interpretation of the spectrum. It is therefore understood that the changes observed in these spectral bands are due to variations in the content of chlorophyll, anthocyanins and carotenoids. As for the final band, it can be observed that in 2024 the region from 920 nm to 970 nm was of upmost importance. Workman & Weyer [18] indicated that this region is related to water absorption (O-H groups). It can therefore be stated that the observed effects in this range are due to changes in the water content of the pant tissue. In 2025, the spectra in that area were more limited, but also an important range can still be seen around 900 nm. Another difference between the two years is that the prominent bands at the extremes lost some importance in 2025, as there were fewer affected plants, while the central bands gained importance. However, due to the similarity of both graphs, it can be concluded that in both the 2024 and 2025 experiments, there was a coincidence in the factors that most affected the plants, which are the variations in pigment and water content.

It is important to point out that Figure 4 also shows negative values in the highlighted ranges. This is to be expected, as this graphic represents the loadings for both PCAs corresponding to vines of the stress group and also of the control group. Each PCA compares the development of the corresponding plant; consequently, for vines that have not exhibited symptoms of water stress, this absence is also reflected. Thus, it has been observed that symptomatic plants exhibit physiological differences due to water stress, which are reflected in the ranges described. However, asymptomatic vines do not exhibit such symptoms and, therefore, do not generate the spectral variations described. Consequently, PCA does not distinguish them based on water stress symptoms. Thus, when evaluating each spectral wavelength in terms of its ability to detect the differences that arise, the wavelengths corresponding to water stress symptoms lose significance.

## 3. Discussion

### 3.1. Stem Water Potential

The potential values obtained throughout the experiment ranged from −0.40 MPa to −0.90 MPa for the stress group, while they varied from −0.40 MPa to −0.65 MPa for the control group. It was observed that when the stressed plants presented values between −0.40 MPa and −0.70 MPa, there were usually no significant differences between groups. However, when the stress group reflected values in the range of −0.70 MPa to −0.90 MPa, significant differences were obtained (Table 1). It could therefore be argued that, based on measurements of stem potential, the detection limit for the effects of water stress lies at around −0.70 MPa. The study conducted by Loggenberg et al. [13] also set the threshold at −0.70 MPa, indicating that vines with values ≥ −0.70 MPa were classified as not under stress. Nevertheless, the study also noted that plants must have values between −1.00 MPa and −1.80 MPa to be classified as stressed. Thus, leaving a range between −0.70 MPa and −1.00 MPa in which vines could not be clearly classified. This is consistent with the point made in Section 2.1, which states that values between −0.70 MPa and −1.00 MPa indicate moderate stress.

### 3.2. HSI Analysis

#### 3.2.1. Spectra

The spectra obtained in this study (Figure 1A,B) are in accordance with those presented by other similar studies [13,14,16,21,22,23,24,25,26,27,28]. This was to be expected, given that these are all typical vegetation spectra in which the visible range (400 nm–700 nm) is influenced by photosynthetic pigments (chlorophyll, carotenoids, anthocyanins, etc.) and the near-infrared (700 nm–1000 nm) by the cellular structure of the leaf (mainly carbohydrates, proteins and leaf water content).

Therefore, there are studies, such as that by Loggenberg et al. [13], that attributed the appearance of the first peak (between 500 nm and 600 nm) to the effect of chlorophyll. However, other studies, such as those by Zovko et al., Nguyen et al. and Ryckewaert et al. [14,16,25], attributed the absorption at 550 nm to the anthocyanin content, while indicating that chlorophyll affected the range between 620 nm and 700 nm. Meanwhile, the study conducted by Yang et al. [28] specified that the greenness index (related to the chlorophyll content) is associated with light absorption at both 554 nm and 677 nm wavelengths.

The valley around 700 nm is located in the boundary between the visible and near-infrared regions, and it is known as the “red edge”. At this edge, as stated by Moghadam et al. and Lowe et al. [24,26], chlorophyll has a high absorption capacity at around 700 nm, but this decreases significantly when passing into the infrared, resulting in a pronounced increase in reflectance.

#### 3.2.2. PCA Results

As shown in Figure 3, there is a clear differentiation between the control plants and those subjected to water stress. This clear separation was also found in other water stress studies, such as those by Loggenberg et al. and Zovko et al. [13,14], in which classification models were applied with accuracies greater than 80% and, in some cases, even 90%. However, a direct comparison of performance between the present study and the cited works is not feasible, owing to differences in experimental design. Specifically, this study involved continuous monitoring and the development of time series analysis to detect a change point or specific day of stress onset, whereas the cited studies relied on single measurements followed by the development of classification models.

Focusing on the most important spectral ranges (Figure 4), the first of these corresponded to the band from 400 nm to 450 nm. The study conducted by Nguyen et al. [16] attributed the effects in this range to chlorophyll degradation, while Ryckewaert et al. [25] stated that the spectral values from 400 nm to 500 nm are related to chlorophyll and carotenoid content. The second band, ranging from 540 nm to 590 nm, is influenced by the effect of pigments such as chlorophyll and anthocyanins. Furthermore, the experiment of Zarco-Tejada et al. [23] also suggested that wavelengths from those bands (515 nm and 570 nm) were related to carotenoid content. The third band was located between 690 nm and 740 nm, coinciding with the aforementioned “red edge” at which, as explained above, chlorophyll is of great importance. There is also research, such as that by Nguyen et al. and Moghadam et al. [16,24], indicating that anthocyanin is related to light absorption at 700 nm. Also, Yang et al. [28] pointed out that the range between 680 nm and 720 nm is related to nitrogen content. Finally, regarding the bands between 920 nm and 970 nm, studies such as those by Zovko et al., Nguyen et al. and Moghadam et al. [14,16,24] suggested that it was related to the canopy water content.

When examining the bands of importance reported in other water stress studies, Loggenberg et al. [13] identified two main ranges. The first, spanning from 475 to 484 nm, aligns with the factors influencing the 400–450 nm region in the present study, specifically, changes in chlorophyll and carotenoid content. In addition, one of the models developed (XGBoost) also showed a region of importance between 624 nm and 708 nm, corresponding to the “red edge”. The results presented by Zovko et al. [14] for the range that matches with this study (400–1000 nm) reflected two significant peaks between 700 nm and 800 nm, coinciding with the “red edge”. The bands of 400–450 nm and 920–970 nm, on the other hand, were not perceived as significant.

#### 3.2.3. Physiological Effects

Considering the above information, the physiological effects that have mainly affected the spectral information and, consequently, the performance of the models generated have been changes in pigment content, such as chlorophyll, carotenoids and anthocyanins, as well as changes in nitrogen and water content. This statement is consistent with the interpretation of the results (Section 2.2.3), and also with the expected physiological symptoms, as they coincide with common symptoms of water stress. In the case of changes in pigments, these are clear indicators of reduced photosynthetic activity, as well as a protective response to oxidative stress [2,14]. The change in nitrogen content, meanwhile, is understood to be a consequence of a variation in the plant protein synthesis, as well as of an accumulation of nitrogenous compounds (amino acids and amines) to act as osmolytes [29]. Finally, it was to be expected that the change in water content was a consequence of a lower absorption by the plant, related to a lower availability in the soil.

#### 3.2.4. Future Research Directions

Future research should focus on validating the predictive window identified in this study (10–13 June) across a broader range of edaphoclimatic conditions and grapevine cultivars to assess its generalizability. A critical next step involves transitioning this approach from controlled potted conditions to commercial vineyards, where environmental heterogeneity and within-canopy variability pose additional challenges. Furthermore, exploring the integration of these hyperspectral signatures with automated data-processing pipelines could enable real-time irrigation decision-support systems. Finally, extending the spectral range beyond 1000 nm or investigating the coupling of VIS–NIR data with thermal imagery may enhance the capacity to detect stress before significant physiological impairment occurs.

## 4. Materials and Methods

### 4.1. Plant Material

Eighteen vines belonging to the cv. Monastrell (*Vitis vinifera* L. ‘Mouvèdre’) were used in this study. These plants were purchased from the Vitis Navarra Nurseries, located in Larraga, Spain (latitude 42°34′4.34″ N, longitude 1°50′52.25″ W and altitude 349 m). These nurseries have indoor facilities for carrying out the grafting process and also fields for both the development of the mother plant material (scions and rootstocks) and for the planting, growth and development of the grafted seedlings. Thus, for this experiment, the plant material consisted of scions of the Monastrell variety (clone 1069) and ‘140 Ruggeri’ and ‘110 Richter’ rootstocks. The grafting was carried out in the spring of 2022, in April, and the seedlings were then planted in the ground until the following January. In January 2023, the plants were acquired by the Public University of Navarre, so they were removed from the ground, transported to the agricultural practices and experimentation rural property of the Higher Technical School of Agricultural Engineering and Biosciences of the Public University of Navarre, located in Pamplona, Spain (latitude 42°47′35.3″ N, longitude 1°37′54.4″ W and altitude 449 m), and planted in pots there.

The climate in Larraga is classified as Mediterranean (Csa) according to Köppen climate classification, with an average rainfall of 572 mm/year and an average temperature equivalent to 13.5 °C for the whole year, 5.5 °C for January (the coldest month) and 22.7 °C for August (the hottest month). In Pamplona, the climate is classified as oceanic (Cf2b), the average rainfall is equivalent to 812.9 mm/year, and the average temperature has a value of 13.3 °C for the whole year, 5.8 °C for January and 22 °C for August [30].

With regard to the materials used for planting, the pots had a capacity of 30 liters and a diameter of 35 cm. A mixture of sand, vermiculite and peat in proportions of 2.5:2.5:1, respectively, was used as growing medium.

The plants were kept in a semi-open tunnel characterized by a roof and front protection but open on the sides. The roof was made of CELLOFLEX 4TT plastic (Riviera Blumen, Murcia, Spain), a multi-layer polyethylene with a light transmission coefficient of over 90% and a diffuse transmission of 15%. Consequently, the vines were subjected to conditions equivalent to those experienced in open air with regard to temperature and relative humidity and very similar with regard to lightning. However, they were shielded from precipitation (rain, hail, snow, etc.). Fertilizers and phytosanitary products were not applied unless strictly necessary.

As explained above, the plants corresponded to two different rootstocks: ‘140 Ruggeri’ (140 R) (8 out of 18 vines) and ‘110 Richter’ (110R) (10 out of 18 vines). It has been demonstrated that these rootstocks have adapted to arid and semi-arid climates, thereby rendering them resistant to drought [31]. To identify each plant, they were assigned a code consisting of the row identification (A for the first row and B for the second), the pot number within each row, and the type of rootstock (e.g., A06 110R).

For this study, all vines were irrigated for a daily total of 28 min, delivered in two 14 min cycles at a rate of 2.3 L/h. This resulted in a total water supply equivalent to 1.07 L/day. The hours assigned for the first and second irrigations were 8:00 a.m. and 4:00 p.m., respectively. The experiment was conducted over two consecutive campaigns, corresponding to the years 2024 and 2025. In 2024, irrigation was discontinued for the vines assigned to the water-stress treatment, starting on 3 June. In 2025, irrigation was not discontinued, so all the vines were irrigated at the same frequency, but for those subjected to stress, the irrigation dose was reduced to 33% (a third of the control dose), starting on 9 June.

In 2024, 8 vines were assigned to the control group and 10 to the stress group, whilst in 2025 half of the plants (9) were assigned to the control group and the remaining half to the stress group. The vines in each group were swapped from one growing season to the next, so that some (but not all) of the vines in the control group in 2024 were moved to the stress group in 2025, and vice versa. The aim was to increase year-to-year variability and make the conditions as close to reality as possible. As the ‘140 Ruggeri’ rootstock is slightly more drought-tolerant and vigorous than the ‘110 Richter’ [31], efforts were made to maintain a balance between the two rootstocks within each group. Consequently, the minimum proportion of each rootstock in each group was set at 33% (a third).

### 4.2. Measurement of Stem Water Potential

Stem water potential (Ψ), defined as the negative pressure under which sap is drawn through the stem xylem, was periodically measured on all vines during the water stress induction period. Measurements were taken using a Scholander-type pressure chamber. For each measurement, a leaf (with its petiole trimmed) was hermetically sealed into the chamber. Nitrogen gas was then progressively injected until xylem sap became visible at the cut end of the petiole. The balancing pressure recorded at this point corresponds to the stem water potential, representing the negative pressure (or tension) under which water is held within the plant vascular system [32].

In all cases, leaves were bagged at 11:00 a.m. and were left for approximately an hour before being cut and inserted into the chamber. This way, measurements were taken at around 12:00 a.m., which corresponds to the time band with the highest levels of solar radiation in June in Pamplona [33]. This method is destructive in nature; consequently, measurements were taken on a weekly basis. During June 2024, data were collected on five dates: the 3rd, 10th, 17th, 21st, and 24th. In June 2025, data were collected on three dates: the 9th, 16th, and 24th.

### 4.3. Image Acquisition and Processing

**Figure 5:**
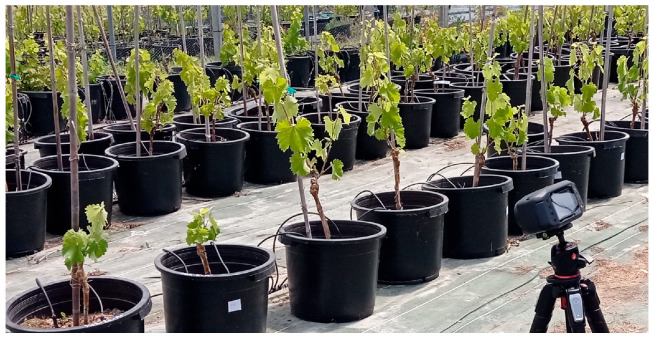
Acquisition of HSI images in the *Vitis vinifera* L. cv. Monastrell pot trial using the Specim IQ camera.

Image acquisition was performed using a Specim IQ push-broom hyperspectral camera (Specim Ltd., Oulu, Finland) mounted on a tripod (Figure 5). The camera operates within the 400–1000 nm range, covering the visible (VIS) and near infrared (NIR) spectral range, with a spectral resolution of 7 nm (204 wavelengths). It is equipped with a CMOS sensor providing a spatial image resolution of 512 × 512 pixels and an integrated RGB camera for reference (5 Mpix) [34].

Images were acquired in the field under natural light conditions between 9:30 a.m. and 11:00 a.m. The equipment (camera and tripod) was transported to the experimental site, and all 18 vines were scanned during each session (Figure 5). Data were collected up to the point at which stem potential measurements indicated the clear presence of water stress. In 2024, this was achieved on the fifth day of imaging, whereas in 2025, as irrigation was not completely halted, the image acquisition period was extended to the seventh day. Consequently, during June 2024, imaging was repeated over five dates: 5th, 7th, 10th, 13th, and 19th. In June 2025, the images were acquired over seven dates: 9th, 11th, 13th, 16th, 18th, 20th, and 24th.

For each acquisition, the camera and tripod were positioned approximately 1.5 m from the plants, providing a field of view of 0.81 × 0.81 m and a spatial resolution of 1.59 mm. The equipment was then turned on and set to automatic integration time. Dark and white references were then acquired. A dark reference, which records sensor noise in the absence of light, was captured automatically. The white reference, representing maximum reflectance, was simultaneously recorded with every sample measurement using a 99% barium sulphate white plate (Specim). All images taken on the same day were obtained under the same conditions, since the exposure time for each image was less than one minute.

The outputs of the camera are two: a hypercube of 512 × 512 × 204 and an RGB image of 512 × 512 × 3. To ensure that both images are correctly aligned, the Specim IQ automatically corrects the parallax between the viewfinder camera and the spectral camera [34].

After acquisition, all images were processed in MATLAB 2024b using custom scripts alongside the Image Processing and Statistics and Machine Learning toolboxes.

**Figure 6:**
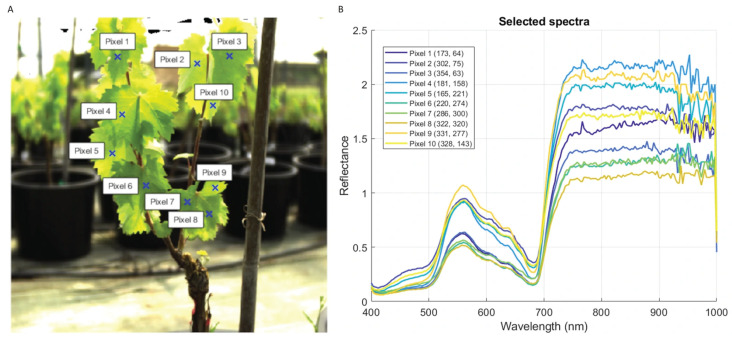
Pixel selection in a hyperspectral image obtained with Specim IQ (**A**); raw reflectance spectra corresponding to the selected pixels (**B**).

In 2024, for each daily RGB image and for each vine, ten pixels were manually selected (blue x) (Figure 6A), and their corresponding spectral data were extracted (Figure 6B). This process generated a dataset of 900 spectra in total (50 pixels per vine). In 2025, with the aim of increasing sample variability, 100 pixels per day and vine were automatically selected. To do so, a mask was manually generated in each image covering as much of the leaf area as possible; once each mask had been created, an automatic and random selection of 100 pixels was made within it. Thus, a total amount of 12,600 spectra (700 pixels per vine) were generated. In both cases, pixels were selected only from the shadowed areas in order to avoid the appearance of defective saturated pixels; also, pixel selection from areas containing leaf veins was avoided.

Then, spectral data were pre-processed per day and vine by a Standard Normal Variate (SNV), smoothing (11 points, polynomial order 2) and mean center to reduce both the scattering and the noise.

### 4.4. Data Analysis

#### 4.4.1. Reference Analysis

The RStudio environment (v.2023.06.1) with R-4.5.2. was used to analyze the data related to stem water potential. First, the data distribution was evaluated using the Kolmogorov–Smirnov test [35,36]. As the data did not follow a normal distribution in this case, the logarithmic transform of the stem water potential variable was performed. Once the transformation was completed, an analysis of variance (ANOVA) was conducted together with a post hoc Duncan test to determine whether there were any significant differences between the treatments [37,38,39].

#### 4.4.2. Spectral Data

From the outset of the experiment, the vines used exhibited inherent differences from one another due to factors such as the rootstock used or variations in growth and development. Consequently, it was decided to carry out spectral analyses of each vine individually, as a combined analysis might have proven much more difficult to interpret. Thus, principal component analysis (PCA) was performed individually on the data from each vine. Subsequently, to analyze the temporal evolution of the signal, the principal component space was used to construct time series based on the baseline conditions of the first day. This was achieved by calculating the Mahalanobis distance of all data points to a reference centroid. For each vine, the centroid was defined by the data corresponding to day 1, and distances were computed for the data points of each subsequent day. The equation used to calculate the Mahalanobis distance was as follows:

(1) $$MD^{2}\left(t\;day,t-1\;day\right)={\left(\mu_{t}-{\bar{\mu }}_{t-1}\right)}^{T}\Sigma REF^{-1}\left(\mu_{t}-{\bar{\mu }}_{t-1}\right),$$

where MD represents the Mahalanobis distance, μ_t_ the scores vector for day t, ${\bar{\mu }}_{t-1}$ the mean vector (centroid) for the first day (t − 1 day) and ΣREF the covariance matrix [40].

To determine whether significant changes occurred over time, the average Mahalanobis distance for each day (t day) was compared with that of the first day (t − 1 day). When, statistically, no significant difference was found between these distances, the data for day t were considered to be similar to the baseline data. Conversely, a significant difference was interpreted as evidence of a notable change in the plant condition between day 1 and day t.

For each vine, it was recorded whether such a change occurred and, if so, the earliest day on which it was detected. Vines showing no significant change were assigned by default to the last day of the trial (day 5 in 2024 and day 7 in 2025). A comparison was made between the results obtained for the control and stress groups, showing both the individual results and the means and standard deviations for each group. However, it should be noted that this comparison was not made using a direct measure as an indicator, but rather a complex derived variable. Thus, the aim was not strictly to verify the existence of statistically significant differences, but rather to evaluate the behavior of each group to see whether they exhibited clear and consistent patterns during both campaigns.

Finally, to identify the sling lengths that contributed most to the PCAs, loadings plots were obtained for the first principal component of each analysis.

## 5. Conclusions

The results obtained in this study demonstrate that the proposed experimental approach, combining the use of a proximal hyperspectral camera with individual PCA analysis, enables the non-destructive detection and characterization of water stress responses of *Vitis vinifera* L. cv. Monastrell vines under controlled pot conditions. Furthermore, the findings reveal a clear relationship between the progression of water stress and the development of pigmentary and structural changes. Thus, changes in the content of photosynthetic pigments (chlorophyll, anthocyanins and carotenoids), nitrogen and water have been found to be the main physiological indicators of water stress. Spectral ranges around 500 nm, 700 nm and 950 nm have been found to be the most informative for early stress detection.

Several limitations should, however, be acknowledged. First, variations in field lighting resulting from the absence of an integrated light source in the sensor required the application of mathematical pre-processing to mitigate these effects. Second, the pot experiment was constrained by a limited sample size.

Notwithstanding these constraints, the findings confirm that hyperspectral imaging holds considerable potential for the detection of water stress in grapevines. Furthermore, if an integration of hyperspectral signatures with automated data-processing pipelines is achieved, real-time irrigation decision-support systems could be developed. Nevertheless, to that end, this method should be validated in commercial vineyards, using wider spectral ranges (beyond 1000 nm) and combining VIS-NIR technology with other techniques, such as thermal imagery, to improve stress detection.

## Figures and Tables

**Table 1:** Stem potential (Ψ) of stress and control vines (*Vitis vinifera* L. cv. Monastrell) during the trial period.

2024	Group	3 June 2024	10 June 2024	17 June 2024	21 June 2024	24 June 2024
	Stress	−0.62 ± 0.09	−0.39 ± 0.06	−0.67 ± 0.08	−0.71 ± 0.18	−0.90 ± 0.09
	Control	−0.64 ± 0.04	−0.42 ± 0.07	−0.42 ± 0.03	−0.41 ± 0.02	−0.61 ± 0.02
	*p*-value	0.275	0.538	<0.01	<0.01	<0.01
	Differences	ns	ns	*	*	*
2025	Group	-	9 June 2025	16 June 2025	-	24 June 2025
	Stress	-	−0.56 ± 0.09	−0.70 ± 0.08	-	−0.90 ± 0.08
	Control	-	−0.54 ± 0.08	−0.64 ± 0.09	-	−0.58 ± 0.07
	*p*-value	-	0.603	0.083	-	<0.01
	Differences	-	ns	ns	-	*

The unit of (Ψ) measurement is MPa. ns: diffences are considered not significant; *: differences are considered significant. Confidence level of 99%, *p*-value = 0.01.

## Data Availability

The original contributions presented in this study are included in the article. Further inquiries can be directed to the corresponding author.

## Abbreviations

The following abbreviations are used in this manuscript:

HSI	Hyperspectral imaging
VIS	Visible
NIR	Near-infrared
SNV	Standard Normal Variate
PCA	Principal component analysis
RF	Random Forest
XGBoost	Extreme gradient boosting
PLS-DA	Partial least squares discriminant analysis
SVMs	Support vector machines
CNN	Convolutional neural network
RGB	Red, green and blue
ANOVA	Analysis of variance

## References

[1] Grainger K., Tattersall H. (2005). Wine Production: Vine to Bottle.

[2] Gu Z., Hu C., Gan Y., Zhou J., Tian G., Gao L. (2024). Role of Microbes in Alleviating Crop Drought Stress: A Review. Plants.

[3] Hanaka A., Majewska M., Jaroszuk-Ściseł J. (2022). Study of the Influence of Abiotic and Biotic Stress Factors on Horticultural Plants. Horticulturae.

[4] Mirás-Avalos J.M., Araujo E.S. (2021). Optimization of Vineyard Water Management: Challenges, Strategies, and Perspectives. Water.

[5] Monteiro E., Gonçalves B., Cortez I., Castro I. (2022). The Role of Biostimulants as Alleviators of Biotic and Abiotic Stresses in Grapevine: A Review. Plants.

[6] Santos R.B., Figueiredo A. (2023). Biotic and Abiotic Stress Management in Grapevine: Recent Advances and Major Breakthroughs. Agronomy.

[7] Swaraj S., Aparna S., Virdee B., Correia S.D., Bedi P., Swaroop A. (2025). Convolutional Neural Networks with Hyperspectral Imaging for Revolutionising Disease Identification and Classification in Agriculture. Proceedings of International Conference on Artificial Intelligence and Networks.

[8] Wu J., Wang J., Wenka H., Feiyan Z., Peiyun W., Chengyi S., Wei G. (2022). Physiology of Plant Responses to Water Stress and Related Genes: A Review. Forests.

[9] Jiang J., Zhou T. (2023). Agricultural drought over water-scarce Central Asia aggravated by internal climate variability. Nat. Geosci..

[10] Burgess A. (2022). Wine without water: Improving grapevine tolerance to drought. Plant Physiol..

[11] Frioni T., Pastore C., Diago M.P. (2023). Editorial: Resilience of grapevine to climate change: From plant physiology to adaptation strategies, volume II. Front. Plant Sci..

[12] Castro Silupu W.M. (2016). Aplicación de la Tecnología de Imágenes Hiperespectrales al Control de Calidad de Productos Agroalimentarios de la Región de Amazonas (Perú). Ph.D. Thesis.

[13] Loggenberg K., Strever A., Greyling B., Poona N. (2018). Modelling Water Stress in a Shiraz Vineyard Using Hyperspectral Imaging and Machine Learning. Remote Sens..

[14] Zovko M., Žibrat U., Knapič M., Kovačić M.B., Romić D. (2019). Hyperspectral remote sensing of grapevine drought stress. Precis. Agric..

[15] Zhang J., Huang Y., Pu R., Gonzalez-Moreno P., Yuan L., Wu K., Huang W. (2019). Monitoring plant diseases and pests through remote sensing technology: A review. Comput. Electron. Agric..

[16] Nguyen C., Sagan V., Maimaitiyiming M., Maimaitijiang M., Bhadra S., Kwasniewski M.T. (2021). Early Detection of Plant Viral Disease Using Hyperspectral Imaging and Deep Learning. Sensors.

[17] Pérez-Roncal C., Arazuri S., Lopez-Molina C., Jarén C., Santesteban L.G., López-Maestresalas A. (2022). Exploring the potential of hyperspectral imaging to detect Esca disease complex in asymptomatic grapevine leaves. Comput. Electron. Agric..

[18] Workman J., Weyer L. (2012). Practical Guide and Spectral Atlas for Interpretive Near-Infrared Spectroscopy.

[19] Humphrey A. (2004). Chlorophyll as a Color and Functional Ingredient. J. Food Sci..

[20] Namitha K.K., Negi P.S. (2010). Chemistry and Biotechnology of Carotenoids. Crit. Rev. Food Sci. Nutr..

[21] Al-Saddik H., Laybros A., Billiot B., Cointault F. (2018). Using Image Texture and Spectral Reflectance Analysis to Detect Yellowness and Esca in Grapevines at Leaf-Level. Remote Sens..

[22] Debnath S., Paul M., Rahaman D.M.M., Debnath T., Zheng L., Baby T., Schmidtke L.M., Rogiers S.Y. (2021). Identifying Individual Nutrient Deficiencies of Grapevine Leaves Using Hyperspectral Imaging. Remote Sens..

[23] Zarco-Tejada P.J., Guillén-Climent M.L., Hernández-Clemente R., Catalina A., González M.R., Martín P. (2013). Estimating leaf carotenoid content in vineyards using high resolution hyperspectral imagery acquired from an unmanned aerial vehicle (UAV). Agric. For. Meteorol..

[24] Moghadam P., Ward D., Goan E., Jayawardena S., Sikka P., Hernandez E. Plant Disease Detection Using Hyperspectral Imaging. Proceedings of the International Conference on Digital Image Computing: Techniques and Applications (DICTA).

[25] Ryckewaert M., Héran D., Trani J.P., Mas-Garcia S., Feilhes C., Prezman F., Serrano E., Bendoula R. (2023). Hyperspectral images of grapevine leaves including healthy leaves and leaves with biotic and abiotic symptoms. Sci. Data.

[26] Lowe A., Harrison N., French A.P. (2017). Hyperspectral image analysis techniques for the detection and classification of the early onset of plant disease and stress. Plant Methods.

[27] Portela F., Sousa J.J., Araújo-Paredes C., Peres E., Morais R., Pádua L. (2024). A Systematic Review on the Advancements in Remote Sensing and Proximity Tools for Grapevine Disease Detection. Sensors.

[28] Yang Z., Tian J., Feng K., Gong X., Liu J. (2021). Application of a hyperspectral imaging system to quantify leaf-scale chlorophyll, nitrogen and chlorophyll fluorescence parameters in grapevine. Plant Physiol. Biochem..

[29] Hossain M.A., Kumar V., Burritt D., Fujita M., Mäkelä P. (2019). Osmoprotectant-Mediated Abiotic Stress Tolerance in Plants: Recent Advances and Future Perspectives.

[30] Fichas Climáticas—Meteo Navarra. https://meteo.navarra.es/climatologia/mapadeestacionesfichas.cfm.

[31] Romero P., Acosta J.M.N., Pérez-Pérez J.G., Dodd I.C., Antolinos V., Marín J.L., Saavedra F.A., Molina E.M., Ordaz P.B. (2025). The rootstock imparts different drought tolerance strategies in long-term field-grown deficit irrigated grapevines. Sci. Hortic..

[32] Scholander P.F., Hammel H.T., Bradstreet E.D., Hemmingsen E.A. (1965). Sap Pressure in Vascular Plants. Science.

[33] Datos de la Estación—Meteo Navarra. https://meteo.navarra.es/estaciones/estacion.cfm?IDestacion=455.

[34] Behmann J., Acebron K., Emin D., Bennertz S., Matsubara S., Thomas S., Bohnenkamp D., Kuska M.T., Jussila J., Salo H. (2018). Specim IQ: Evaluation of a New, Miniaturized Handheld Hyperspectral Camera and Its Application for Plant Phenotyping and Disease Detection. Sensors.

[35] Fasano G., Franceschini A. (1987). A multidimensional version of the Kolmogorov—Smirnov test. Mon. Not. R. Astron. Soc..

[36] Lopes R.H.C., Reid I.D., Hobson P.R. (2007). The two-dimensional Kolmogorov-Smirnov test. Proceedings of the XI International Workshop on Advanced Computing and Analysis Techniques in Physics Research, Amsterdam, The Netherlands, 23–27 April 2007.

[37] Adeniran A.T., Olilima J.O., Akano R.O. (2021). Analysis of Variance: The Fundamental Concepts and Application with R. Int. J. Math. Comput. Res..

[38] Tallarida R.J., Murray R.B., Tallarida R.J., Murray R.B. (1987). Duncan Multiple Range Test. Manual of Pharmacologic Calculations: With Computer Programs.

[39] Mooi E., Sarstedt M., Mooi-Reci I., Mooi E., Sarstedt M., Mooi-Reci I. (2018). Hypothesis Testing & ANOVA. Market Research: The Process, Data, and Methods Using Stata.

[40] De Maesschalck R., Jouan-Rimbaud D., Massart D.L. (2000). The Mahalanobis distance. Chemom. Intell. Lab. Syst..

